# Sustainable Active Packaging Based on Chitosan–Pomegranate Peel Biofilm Protects Pecorino Romano PDO Cheese Through Polyphenol-Driven Antioxidant and Antimicrobial Activity

**DOI:** 10.3390/molecules31152631

**Published:** 2026-07-28

**Authors:** Sara Palmieri, Valentina Mangano, Elisa Capini, Giulia De Grazia, Giovanna Loredana La Torre, Federico Fanti, Manuel Sergi, Andrea Salvo

**Affiliations:** 1 Department of Biosciences and Technologies for Agriculture, Food and Environment, University of Teramo, Via Balzarini 1, 64100 Teramo, Italy; spalmieri@unite.it (S.P.); ffanti@unite.it (F.F.); 2 AEDES S.r.l., Via Cancelliera 12, 00041 Albano Laziale, Italy; valentina-mangano@libero.it (V.M.); elisa.carpini@libero.it (E.C.); giulia.degrazia@aedes.info (G.D.G.); 3 Department of Biomedical and Dental Sciences and Morpho-Functional Imaging, University of Messina, Polo Universitario Annunziata, Viale Annunziata, 98168 Messina, Italy; 4 Department of Chemistry and Drug Technology, University of Rome La Sapienza, Via P. le A. Moro 5, 00185 Roma, Italy; manuel.sergi@uniroma1.it

**Keywords:** chitosan, edible biofilm, pomegranate peel, polyphenols, active packaging, shelf-life, food safety

## Abstract

A chitosan-based edible biofilm enriched with pomegranate peel extract and essential oils was developed as a sustainable active packaging material that exploits agro-industrial by-products, in agreement with circular economy principles. Targeted HPLC–MS/MS analysis quantified twelve polyphenols, with total polyphenol content ranging from 88.20 to 137.38 μg/g dry weight. Ellagic acid was the predominant compound (90.78 μg/g dry weight), followed by gallic acid (28.80 μg/g dry weight). Untargeted HPLC–HRMS analysis revealed additional flavonoid and ellagitannin-related derivatives, confirming the chemical complexity of the incorporated phenolic fraction. ICP-OES analysis showed heavy metal concentrations below the analytical quantification limits, supporting compliance with food-contact safety requirements. During 45 days of refrigerated storage, coated Pecorino Romano PDO cheese exhibited lower yeast and mold counts than uncoated controls, with a maximum reduction of approximately 0.87 log_10_ CFU/g. Enterobacteriaceae remained below the detection limit in coated samples throughout storage, whereas they were detected in untreated cheese at the end of storage. These findings demonstrate the potential of the developed edible biofilm as a safe and sustainable active packaging material for refrigerated dairy products while promoting the valorization of pomegranate processing by-products.

## 1. Introduction

The adoption of biotechnological strategies in food packaging has markedly accelerated the development of intelligent materials with enhanced functional properties, capable of improving microbiological safety, sensory quality, and shelf life of food products [1]. Furthermore, increasing environmental concerns associated with the extensive use of petroleum-based packaging materials have intensified the search for biodegradable, renewable, and sustainable alternatives capable of reducing plastic waste while maintaining food quality and safety. Among the strategies currently under investigation, edible films based on natural biopolymers have emerged as one of the most promising solutions, as they combine biodegradability, the absence of undesirable residues, environmental compatibility, and the ability to incorporate bioactive compounds with antimicrobial and/or antioxidant properties [1,2]. In this context, research has increasingly focused on film-forming matrices derived from polysaccharides, such as starches, alginates, and chitosan, which can generate mechanically stable structures and can be further functionalized using plant-derived biomolecules [3].

Among these materials, chitosan, obtained by deacetylation of chitin, has attracted considerable attention due to its favorable technological and functional properties. Its high film-forming capacity, together with the presence of protonatable amino groups that confer a positive charge under acidic conditions, underpins its well-documented antimicrobial activity against Gram-positive and Gram-negative bacteria, as well as yeasts and molds [4,5]. This antimicrobial activity has been attributed to multiple mechanisms, including electrostatic interactions with microbial cell membranes, disruption of membrane permeability, metal ion chelation, and inhibition of key enzymatic systems [6,7]. Owing to these characteristics, chitosan is a particularly suitable candidate for formulating active biopolymeric coatings for high–added–value food products.

Concurrently, increasing attention has been devoted to recovering polyphenol–rich extracts from agri-food by-products, in accordance with sustainability and circular economy principles. Pomegranate peel (*Punica granatum* L.), commonly discarded during industrial processing, is recognized as an exceptionally rich source of phenolic compounds, including ellagitannins, ellagic and gallic acids, catechins, and glycosylated flavonoids [8]. Notably, pomegranate peel represents approximately 30–50% of the total fruit weight and is frequently discarded during juice and food processing, generating substantial quantities of agro-industrial waste. These bioactive molecules have been widely reported to exhibit antioxidant, antimicrobial, metal-chelating, and selective pro-oxidant activities, contributing effectively to food preservation and the control of spoilage-associated microflora [9,10].

Besides their individual biological activities, chitosan and pomegranate peel-derived polyphenols may exert complementary and synergistic effects when incorporated into the same polymeric matrix. Chitosan primarily acts by disrupting microbial cell membranes through electrostatic interactions and by chelating essential metal ions, whereas polyphenols contribute through radical scavenging activity, inhibition of oxidative degradation, interactions with microbial proteins and membranes, and modulation of intracellular redox balance. In addition, the incorporation of food-grade essential oils may further enhance membrane permeabilization and facilitate the diffusion of phenolic compounds, thereby improving the overall antimicrobial effectiveness of the edible film. Such complementary mechanisms have increasingly been recognized as the basis for multifunctional active packaging systems combining antioxidant protection with antimicrobial activity and extended food preservation [11,12]. This synergistic behavior constitutes the scientific rationale underlying the patented chitosan–pomegranate peel edible biofilm investigated in the present study, which was specifically designed to combine antimicrobial protection, antioxidant functionality, and sustainability within a single active packaging system.

Within the dairy sector, long-ripened cheeses such as Pecorino Romano PDO constitute suitable matrices for active packaging applications, as their surfaces are particularly susceptible to contamination by environmental microorganisms during storage and maturation, potentially resulting in sensory defects and reduced shelf life [13]. The application of antimicrobial surface coatings represents an effective approach to reducing secondary contamination while maintaining the intrinsic physicochemical and sensory properties of the treated food. Nevertheless, natural edible coatings must be carefully characterized for elemental safety, material stability, release behavior of bioactive compounds, and antimicrobial performance under realistic storage conditions [14].

Although several studies have investigated chitosan-based films incorporating different plant-derived bioactive compounds (Table 1), limited information is available regarding the comprehensive chemical characterization, chemical safety assessment, and long-term microbiological performance of patented chitosan-pomegranate peel biofilms applied to Pecorino Romano PDO cheese under refrigerated storage conditions.

Furthermore, studies integrating targeted and untargeted polyphenolic characterization with microbiological monitoring and food-contact safety assessment remain scarce. Therefore, further research is required to evaluate the effectiveness and safety of multifunctional biofilm in real food systems. Within this context, this study aimed to develop, chemically characterize, and evaluate the microbiological performance of a patented chitosan-based edible biofilm enriched with pomegranate peel extracts and essential oils for application as an active coating on *Pecorino Romano PDO* cheese during refrigerated storage. The patented technology [23] was applied as a functional coating on *Pecorino Romano PDO* cheese and evaluated during a 45-day storage period. An integrated analytical approach was adopted, including: (i) ICP–OES for elemental safety assessment; (ii) targeted analysis by HPLC–MS/MS for the quantification of polyphenols; (iii) untargeted HPLC–HRMS profiling; and (iv) longitudinal microbiological analyses on the film and on coated and uncoated cheese samples.

In order to protect the intellectual property associated with the patented formulation, selected compositional ratios and processing parameters are not fully disclosed. Nevertheless, all methodological steps relevant to interpreting the experimental workflow and evaluating biofilm performance are reported in sufficient detail.

## 2. Results and Discussion

### 2.1. Physicochemical Properties and Mechanical Performance of the Biofilm

The chitosan–pomegranate peel edible films developed in this study underwent physicochemical and mechanical characterization to assess their suitability for food-contact applications. Physicochemical analyses were conducted in compliance with European Parliament Regulation (EC) No 1935/2004 [24], European Commission Regulation (EU) No 10/2011 [25], and European Commission Regulation (EC) No 2023/2006 [26] on good manufacturing practice for food-contact materials, ensuring adherence to MOCA safety requirements.

Physicochemical analyses and mechanical evaluations available for the patented formulation were carried out according to standardized procedures commonly adopted for food-contact films and biopolymeric materials, including UNI EN ISO 527-3 [27], UNI EN ISO 527-1 [28], and ASTM D618-21 [29].

Mechanical testing demonstrated satisfactory structural integrity and flexibility of the patented biofilm. Tensile strength ranged from 1.17 ± 0.58 to 1.21 ± 0.22 MPa, elongation at break ranged from 29.7 ± 1.7% to 32.3 ± 1.2%**,** and elastic modulus ranged from 3.9 ± 0.05 to 4.1 ± 0.07 MPa. These results indicate adequate deformability and resistance under tensile stress, supporting the intended application of the material as an edible food-contact coating [23]. Scanning electron microscopy (SEM) provided complementary morphological characterization of the biofilm and further supported the mechanical evaluation. Examination of the fracture surfaces revealed a compact and continuous matrix with a homogeneous thickness of approximately 240 μm. The fractured sections maintained structural continuity without evidence of extensive cracking, delamination, or phase separation phenomena. The observed morphology suggested a flexible and elastic structure, consistent with the tensile properties reported for the patented biofilm formulation [23]. Furthermore, the hydrated appearance of the matrix was consistent with the presence of water within the chitosan–polyphenol network, which may contribute to the plasticization behavior and structural stability during handling and application.

The developed biofilms also exhibited satisfactory handling properties and physical stability during application and storage. Incorporation of pomegranate peel–derived phenolic compounds into the chitosan matrix did not compromise film integrity. Similar findings have been reported for chitosan-based films enriched with phenolic-rich natural extracts, where intermolecular interactions between chitosan chains and polyphenols contribute to structural stabilization and improved film performance [30,31].

From a safety perspective, all measured parameters complied with the applicable regulatory requirements. The main physicochemical characteristics of the films were: pH 3.70; density 1.022 g/mL; total wet-soluble matter 82.27 ± 5.20%; and total dry-soluble matter 21.67 ± 4.68%. Phthalates, lactose, and gluten were not detected.

Overall, the formulated biofilm was free from common allergenic and toxic contaminants and exhibited physicochemical and mechanical properties compatible with food-contact applications. The tensile properties of the developed biofilm are consistent with previous studies on chitosan-based films containing phenolic-rich plant extracts, where intermolecular hydrogen bonding and electrostatic interactions between chitosan and polyphenols contribute to structural stabilization and preservation of film integrity [30,31,32].

### 2.2. Elemental Composition (ICP–OES)

The determination of inorganic elements was performed because the developed biofilm was specifically designed for food-contact applications, and the assessment of potentially toxic elements represents a mandatory safety parameter under current European regulations on materials intended for food contact. In this context, inductively coupled plasma optical emission spectrometry (ICP–OES) was employed to evaluate the presence of arsenic (As), lead (Pb), cadmium (Cd), and mercury (Hg), which are non-essential elements known to exert toxic effects even at low exposure levels [33,34].

Potential contamination by these elements may originate from the raw materials used for biofilm production, including pomegranate peel, as well as from agricultural practices, environmental exposure, or processing and handling conditions. The uptake and accumulation of heavy metals in plant-derived matrices are well documented and strongly influenced by soil physicochemical characteristics, irrigation water quality, and anthropogenic activities [35]. Chronic intake above the tolerable limits may result in severe adverse health effects, including neurological alterations; cadmium exposure, in particular, has been associated with hypertension, renal and cardiovascular dysfunctions, skeletal damage, reproductive toxicity, and potential carcinogenic effects [33].

The toxicological relevance of As, Cd, Hg, and Pb is commonly assessed through health-based guidance values established by international authorities. Table 2 summarizes the tolerable weekly intake (TWI) values proposed by JECFA [36] and FAO/WHO [37]. These values refer to human dietary exposure and are provided solely as toxicological reference points; they do not represent regulatory limits for food-contact materials.

In addition, according to Directive 94/62/EC [39] on packaging and packaging waste, the total concentration of Pb, Cd, Hg, and Cr (VI) in packaging materials must not exceed 100 ppm. In the present study, all the investigated elements were below the quantification limits of the analytical method. Even if no specific migration or content limit is defined for arsenic in food-contact materials, its toxicological relevance is addressed through the establishment of health-based guidance values, including the TWI reported and set at 0.015 mg per kg of body weight.

Sample preparation and elemental determination were performed in accordance with UNI EN 13805 [40] for pressure-assisted digestion and UNI EN ISO 11885 [41] for ICP–OES analysis. Method validation demonstrated excellent analytical performance, with high linearity (coefficients of determination consistently higher than 0.99987), satisfactory accuracy and precision, and low limits of detection (LOD) and quantification (LOQ) for all investigated elements (Table 3).

The recovery obtained for Cd (110.99%) remained within the acceptance range generally considered suitable for trace elemental analysis and may reflect minor matrix-enhancement effects associated with the digestion procedure and instrumental determination. The corresponding precision (RSD = 3.8%) confirmed the robustness of the analytical method. As Table 4 shows, all toxic investigated elements were below the method detection limits (LOD) and therefore could not be quantitatively determined. These findings indicate the absence of measurable contamination by As, Cd, Hg, and Pb in the developed biofilm.

Consequently, the total content of As, Pb, Cd, and Hg was far below the cumulative limit of 100 ppm established by Directive 94/62/EC [39]. Although arsenic is not included within this regulatory limit, it was likewise below the analytical detection limit. Furthermore, elemental analysis revealed that the edible films contained only trace levels of essential elements (Fe, Zn, Cu, Mn), all well below regulatory thresholds for food-contact materials [42].

Overall, the elemental concentrations detected in the biofilm were below the validated analytical method’s limits of detection, indicating the absence of measurable contamination by the investigated toxic elements.

The absence of toxic metals also confirms that any antimicrobial activity observed in subsequent microbiological assays cannot be attributed to metal contamination, which is known to exert bactericidal effects under certain conditions [43], but rather to the intrinsic and synergistic action of chitosan and polyphenolic compounds derived from pomegranate peel.

### 2.3. Polyphenolic Profile of the Biofilm: Targeted HPLC–MS/MS Quantification and Untargeted HPLC–HRMS Analysis

The antioxidant and antimicrobial properties of the developed biofilm are influenced not only by the intrinsic characteristics of chitosan but also by the presence of phenolic compounds incorporated from pomegranate peel powder. To better characterize the presence and levels of these bioactive constituents, the polyphenolic profile of the biofilm was investigated using targeted HPLC–MS/MS analysis of 36 analytes, complemented by an untargeted HPLC–HRMS approach for broader compound screening.

#### 2.3.1. Targeted HPLC–MS/MS Analysis

A targeted qualitative and quantitative analysis was achieved using authentic standards, enabling the identification and quantification of twelve polyphenolic compounds. Six biofilm subsamples (TQ1–TQ6) were collected from different areas of the biofilm surface to assess spatial homogeneity and were analyzed without dilution.

The quantitative results (Table 5) revealed a highly consistent polyphenolic profile across all subsamples. The quantified total polyphenol content (sum of the twelve detected compounds) ranged from 88.20 to 137.38 μg/g dry weight (dw), confirming the effective incorporation of phenolic compounds into the biofilm matrix. Variability among sampling points was limited and within the expected analytical deviation, supporting homogeneous dispersion of the peel-derived fraction during film casting.

Ellagic acid was the predominant compound in all samples, reaching concentrations up to 90.78 μg/g dw in Biofilm TQ6 and not falling below 55.99 μg/g dw in the remaining subsamples. When expressed as a percentage of total quantified polyphenols, ellagic acid accounted for approximately 60–70%, confirming its dominant contribution. This finding is consistent with previous studies identifying ellagic acid as a major hydrolysable tannin derivative in pomegranate peel [44,45].

Gallic acid was the second most abundant phenolic acid, accounting for ≈18–22% of the quantified compounds, with concentrations up to 28.80 μg/g dw. Quercetin hexoside was detected at 10.69–15.40 μg/g dw, corresponding to approximately 8–12% of the total phenolic fraction. Minor flavonoids, including epicatechin, apigenin, and naringenin, were consistently quantified at concentrations below 1% of total phenolics, while syringic acid and chlorogenic acid were present at trace levels (<0.05 μg/g dw).

The variability among subsamples was limited, indicating effective dispersion of the peel-derived fraction during film preparation. A slightly lower apigenin in biofilm TQ4 likely reflects localized sampling variation rather than compositional heterogeneity.

Although caffeic acid was included among the targeted analytes, it was not detected in any subsample. Compared with literature values reported for raw pomegranate peel [45,46], the concentrations observed in the biofilm were lower, which can be reasonably attributed to (i) dilution within the chitosan matrix, and (ii) partial degradation of thermolabile compounds during freeze-drying and thermal processing steps [44].

From a mechanistic standpoint, the predominance of hydrolysable tannin-related compounds, particularly ellagic and gallic acids (>60% of total quantified phenolics), provides a compositional basis for the antioxidant potential of the developed matrix [44,45,46]. Similar chitosan-based films enriched with pomegranate peel extracts exhibited significant increases in total phenolic concentration and radical scavenging activity, consistent with previous studies demonstrating that phenolic-rich matrices enhance oxidative stability in packaged foods [47,48]. In addition, flavonol derivatives, such as quercetin and myricetin, may further contribute to antimicrobial activity [49].

Therefore, the preservation of the phenolic compounds within the developed matrix indicates that the fabrication conditions did not substantially compromise the bioactive profile of the peel-derived extract, supporting its potential as an effective active packaging system.

#### 2.3.2. Untargeted HPLC–HRMS Analysis and Putative Identification

To complement the targeted HPLC–MS/MS quantification and to further characterize the biofilm, an untargeted HPLC–HRMS analysis was performed. The high-resolution approach aimed to expand compound annotation beyond the twelve target analytes and provide a broader chemical fingerprint within the chitosan matrix.

Because suitable internal standards were not available for the additional features detected, compound identification was considered putative, based on accurate mass, isotopic pattern, chromatographic behavior, the literature data, and available databases. The compounds tentatively identified through this untargeted approach are summarized in Table 6. Identifications are putative (see Section 3.7); no quantification was performed.

The HPLC–HRMS analysis confirmed the presence of all twelve compounds previously detected by targeted HPLC–MS/MS analysis and revealed additional features associated with ellagitannin-related structures. In particular, ions detected at *m*/*z* 781, 933, and 1085 were consistent with high-molecular-weight ellagitannin derivatives typically reported in pomegranate peel extracts. Accurate mass deviations were below 5 ppm, and isotopic pattern matching exceeded 85%, supporting reliable elemental composition assignment.

Several chromatographic peaks sharing identical accurate masses were observed, suggesting the presence of positional or structural isomers, which are commonly reported for ellagitannin and flavonoid-derived compounds in pomegranate matrices. These isomeric features were differentiated by their retention times but could not be unambiguously resolved without reference standards.

Overall, the untargeted screening revealed a broader phenolic diversity, including hydrolysable tannins, low-molecular-weight phenolic acids, flavonoid derivatives, and glycosylated conjugates, suggesting that the extraction and film-forming process preserved a substantial portion of the native phenolic complexity of pomegranate peel. However, the absence of authentic standards precluded definitive structural confirmation and discrimination among certain positional isomers.

Despite these limitations, the untargeted results are consistent with the targeted quantification data and support a homogeneous dispersion of phenolic constituents within the chitosan matrix. The coexistence of hydrolysable tannins, simple phenolic acids, and flavonols suggests complementary antioxidant mechanisms, including hydrogen atom transfer (HAT), single-electron transfer (SET), and metal chelation, consistent with the antioxidant activity observed for the developed biofilm.

Putative identifications; no quantification was performed. Taken together, the combined targeted and untargeted analytical approaches provide a chemically coherent rationale for describing the developed material as a phenolic-enriched multifunctional biofilm. No evidence of major structural modification or degradation of the principal phenolic constituents was observed under the experimental conditions, consistent with previous reports on chitosan–phenolic composite systems [50].

### 2.4. Microbiological Results

The antimicrobial performance of the developed chitosan–polyphenol biofilm was evaluated by monitoring microbial evolution of both the film in its original form and in Pecorino Romano PDO cheese, either uncoated (control) or coated with the biofilm, throughout 45 days of refrigerated storage (T0–T3). Results are summarized in Table 7 and Table 8.

The edible biofilm exhibited negligible microbiological quality at the beginning of storage (Table 7), with total viable counts (TVC, 30 °C) and yeast and mold counts below the detection limit (0.00 log_10_ CFU/g). During storage, TVC increased to 2.17 log_10_ CFU/g after 360 h, then decreased to 1.54 log_10_ CFU/g at 1080 h. Fungal counts remained below 1 log_10_ CFU/g throughout the storage period and returned to non-detectable levels at T3. These findings demonstrated the good microbiological stability of the biofilm matrix and its limited susceptibility to microbial colonization. Such behavior is consistent with the well-documented antimicrobial properties of chitosan, whose polycationic nature is known to interfere with microbial cell membranes and reduce microbial adhesion to polymeric surfaces [51,52].

In uncoated Pecorino Romano PDO (Table 8), TVC increased progressively from 3.48 log_10_ CFU/g at T0 to 6.45 log_10_ CFU/g after 360 h and remained at comparable levels throughout refrigerated storage, reaching 6.80 log_10_ CFU/g at T3. Yeasts and molds showed an initial population of 3.52 log_10_ CFU/g, decreased to 2.43 log_10_ CFU/g at T1, and subsequently remained within the range of 3.01–3.09 log_10_ CFU/g, reflecting the normal evolution of the surface microbiota during storage of ripened hard cheeses, where oxygen availability, moisture redistribution, and nutrient diffusion influence microbial succession [53].

Coated cheese samples (Table 8) showed a similar increase in TVC during storage, from 3.51 log_10_ CFU/g at T0 to 6.13 log_10_ CFU/g at T3. Differences between coated and uncoated cheese of total bacterial counts remained limited (<0.7 log units at all time points), suggesting that the complex bacterial consortium naturally associated with ripened cheese was only marginally affected by the edible coating.

In contrast, a more pronounced effect was observed against yeasts and molds. At 360 h of storage, coated samples exhibited fungal counts of 1.56 log_10_ CFU/g compared with 2.43 log_10_ CFU/g in the control, corresponding to a reduction of approximately 0.87 log units (≈87%). Therefore, the early reduction suggests that chitosan-polyphenol coating may delay visible spoilage, reduce product losses associated with surface contamination, and help to extend the commercial shelf life of aged cheeses. At 720 h, coated samples still showed significantly lower fungal counts than the controls, whereas after 1080 the differences progressively decreased, indicating a gradual attenuation of the antifungal activity during prolonged storage.

**Figure 1:**
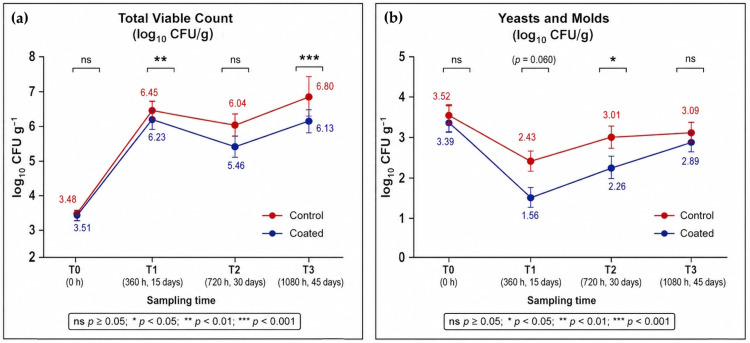
Microbiological trends of uncoated (Control) and coated (Coated) Pecorino Romano PDO cheese during refrigerated storage. (**a**) Total variable count (log_10_ CFU/g). (**b**) Yeasts and molds (log_10_ CFU/g). Values are expressed as mean ± standard deviation (*n* = 3). Statistical comparison between coated and control samples at each sampling time was performed using Welch’s *t*-test. ns *p* ≥ 0.05; * *p* ≤ 0.05; ** *p* ≥ 0.01; *** *p* ≤ 0.001.

Therefore, as Figure 1 shows, for Pecorino Romano PDO cheese, total viable counts increased progressively during refrigerated storage in both coated and uncoated samples, reflecting the normal evolution of the cheese microbiota. Although both groups exhibited similar trends, coated samples generally showed lower bacterial populations than the untreated controls. Statistically significant differences were observed at T1 (*p* < 0.01) and T3 (*p* < 0.001), whereas no significant differences were detected at T0 and T2 (*p* > 0.05). The maximum reduction achieved was approximately 0.67 log_10_ CFU/g at the end of storage, suggesting that the biofilm moderately limited bacterial proliferation without significantly altering the natural microbial dynamics of the cheese.

More pronounced effects were observed against yeasts and molds, which represent the principal spoilage microorganisms on the cheese surface during refrigerated storage. Initial fungal counts did not differ significantly between coated and uncoated cheese (*p* > 0.05), confirming the comparability of the experimental groups before treatment. During storage, coated samples consistently exhibited lower fungal populations than the controls, reaching reductions of approximately 0.87 and 0.75 log_10_ CFU/g at T1 and T2, respectively. The difference was statistically significant at T2 (*p* < 0.05), whereas a strong inhibitory trend was observed at T1 despite the lack of statistical significance, probably reflecting the biological variability associated with microbial enumeration. At the end of storage, fungal counts remained lower in coated samples, although the difference was no longer statistically significant.

Enterobacteriaceae were detected exclusively in untreated cheese at 720 and 1080 h (1.8–2.4 log_10_ CFU/g, respectively), whereas coated samples remained below the detection limit throughout the refrigerated storage period. Although the microbial levels observed in the control samples were relatively low, the absence of detectable Enterobacteriaceae in coated cheese is technologically relevant because these microorganisms are widely recognized as indicators of hygienic quality and have been associated with undesirable proteolysis, gas formation, and off-flavor development in hard cheeses [53].

The selective inhibition of fungal populations observed in the present study is consistent with the antimicrobial mechanisms previously described for chitosan-based edible coating [51,54]. Moreover, the phenolic profile identified by UHPLC-HRMS analysis may have contributed to the observed antimicrobial behavior. Ellagic acid, the predominant phenolic constituent of the biofilm, together with gallic acid and quercetin hexoside derivatives, has been widely reported to interfere with fungal cell membrane integrity, increase membrane permeability, and disrupt intracellular enzymatic processes. Although the present study was not designed to establish a direct quantitative relationship between the concentration of individual phenolic compounds and microbial inhibition, the abundance of these phenolics is consistent with the selective antifungal activity observed during cheese storage. The antimicrobial performance of the coating is therefore likely attributable to the combined action of chitosan and the naturally occurring phenolic constituents extracted from chestnut by-products, whose complementary mechanisms may enhance fungal growth inhibition [50,55].

Overall, the chitosan–polyphenol biofilm did not prevent the progressive increase in total bacterial population naturally associated with *Pecorino Romano PDO* cheese during storage. However, the coated samples consistently exhibited lower yeast and mold populations than the untreated control, particularly during the first weeks of storage, when fungal spoilage is most critical for product preservation. These findings support the potential application of the developed coating as a natural antifungal barrier for the surface protection of ripened cheeses, and are consistent with previous studies reporting greater efficacy of chitosan-based edible coatings against fungal contaminants than against complex bacterial communities associated with dairy products [50,52].

## 3. Materials and Methods

### 3.1. Samples and Chemicals

Pecorino Romano PDO cheeses were purchased from dairy retailers located in Rome (Italy).

Chitosan was obtained from Mystic Moments (Fordingbridge, UK; 19/20 Sandleheath Industrial Estate); the product was 100% natural and GMO-free. Essential oils were produced by steam distillation of the aerial parts of *Thymus vulgaris*, *Citrus limon*, and *Laurus nobilis*, as well as from propolis; they were supplied by Esperis S.p.A. (Milan, Italy) and stored at 4 °C until use.

Granular polyvinyl alcohol (PVA) was purchased from Alquera (Madrid, Spain), and vegetable glycerol was obtained from Carlo Erba (Milan, Italy).

For ICP–OES analyses, hydrochloric acid (37%) and nitric acid (68%) were purchased from Merck (Darmstadt, Germany). Deionized water was produced using a Millipore Supelco purification system (Milan, Italy). Single-element standard solutions (1000 mg/L in 2% nitric acid) of Hg, As, Cd, and Pb, at the highest available purity, were also obtained from Merck (Darmstadt, Germany).

For polyphenol determination, the following analytical grade standards (≥98% purity) were purchased from Vinci BioChem (Vinci, Florence, Italy): gallic acid, hydroxytyrosol, protocatechuic acid, (−)-epigallocatechin, 3-hydroxybenzoic acid, tyrosol, chlorogenic acid, epicatechin, caffeic acid, vanillic acid, catechin, (−)-epigallocatechin gallate, syringic acid, orientin, rutin, *p*-coumaric acid, hyperoside, isoquercitrin, ferulic acid, hesperidin, rosmarinic acid, oleuropein, *o*-coumaric acid, sinapic acid, myricetin, luteolin, quercetin, *trans*-cinnamic acid, naringenin, isoxanthohumol, apigenin, diosmetin, kaempferol, xanthohumol, ellagic acid, and delphinidin-3-glucoside.

Ultrapure water, acetic acid, and methanol, all UPLC-MS grade, were purchased from Scharlab (Lodi, Italy). Working standard mixtures were prepared by appropriate dilution in methanol and stored at −20 °C until analysis.

### 3.2. Pomegranate Peel Treatment

Approximately 1 kg of pomegranate peels was obtained as an agro-industrial by-product from a local company involved in pomegranate juice production. The material consisted of peels remaining after juice extraction and was supplied already sun-dried by the producer. Because the raw material originated from industrial processing, information regarding cultivar, harvesting period, maturity stage, and geographical origin was not available.

The dried peels were homogenized using a laboratory blender and subsequently frozen at −80 °C using an Alpha 2–4 LD plus CHRIST freeze dryer. The lyophilized material was further ground and sieved to obtain a homogeneous powder with an average particle size of approximately 0.5 mm.

An aqueous extraction was then performed by suspending 30 g of the lyophilized pomegranate peel powder in 100 mL of distilled water. The suspension was heated to boiling (approximately 100 °C, at atmospheric pressure) on a thermostatically controlled hot plate under continuous magnetic stirring, and maintained under gentle boiling for 40 min. After cooling to room temperature, the extract was filtered through a 0.45 μm paper filter, collected in amber glass bottles, sealed, and stored at 4 °C until incorporation into edible film formulation.

This extraction procedure was adopted to recover the water-soluble bioactive fraction of pomegranate peel while preserving compatibility with the food-grade formulation of the patented biofilm.

### 3.3. Preparation of Edible Films

Edible films were prepared according to a patented formulation previously described by Salvo et al. [23] and subsequently applied in related studies [1,2]. To preserve the proprietary aspects of the patented technology, selected compositional ratios and specific processing parameters are not fully disclosed.

Food-grade chitosan, characterized by a molecular weight and degree of deacetylation suitable for antimicrobial film formation, was employed as the primary film-forming polymer. Chitosan was dissolved in an aqueous acetic acid solution under controlled heating and continuous magnetic stirring until complete solubilization. The acid concentration and selected formulation parameters constitute proprietary information protected by the patent and therefore cannot be disclosed. However, the dissolution conditions were optimized to obtain a homogeneous polymer suitable for film formation. Food-grade polyvinyl alcohol (PVA), previously dissolved in distilled water, was incorporated into the chitosan solution to improve film cohesion and mechanical stability.

Food-grade vegetable glycerol was then added as a plasticizing agent to improve flexibility and reduce the polymeric matrix brittleness. Subsequently, the aqueous pomegranate peel extract was incorporated under controlled stirring conditions to ensure homogeneous dispersion of the phenolic fraction throughout the film-forming solution.

A mixture of food-grade essential oils (*Thymus vulgaris*, *Citrus limon*, *Laurus nobilis*) together with propolis extract was finally incorporated under continuous stirring at ambient temperature to provide additional antimicrobial functionality.

The resulting film-forming solution was cast onto inert glass substrates using a calibrated chromium-plated steel film applicator (Wasag film applicator, model 288; Erichsen GmbH & Co. KG, Hemer, Germany). After casting, the films were dried under controlled laboratory conditions at 25 ± 2 °C (for 48 h) and a relative humidity (RH) of 50 ± 5% until complete physical stabilization was achieved. The dried films were carefully removed from the casting plates and conditioned according to ASTM D618-21 [29] before further characterization.

The morphology and cross-sectional structure of the films were evaluated by scanning electron microscopy (SEM), which also allowed estimation of the film thickness (approximately 240 μm). For each formulation, five films were prepared per batch, and three independent batches were produced to verify the reproducibility of the manufacturing process.

Additional details regarding the exact formulation and selected processing parameters are not disclosed to preserve the intellectual property associated with the patented technology.

### 3.4. ICP–OES Elemental Analysis

The elemental composition of the chitosan–pomegranate peel edible films was determined by inductively coupled plasma optical emission spectrometry (ICP–OES) to evaluate the presence of potentially toxic metals and essential trace elements. The analytical procedure was adapted from validated protocols established for food-contact materials [56]. For each analysis, approximately 200 mg of dried film samples were precisely weighed and subjected to acid digestion in TFM (a modified PTFE with thermoplastic parts for a homogeneous, non-porous surface that avoids contamination and memory effects) vessels by the addition of 5 mL of concentrated nitric acid (HNO_3_, 65% *v*/*v*).

Sample mineralization was achieved using a closed-vessel microwave digestion system (Ethos UP, Milestone) with a controlled-temperature program consisting of a ramp to 160 °C for 10 min, followed by a 20 min holding step at that temperature. After digestion, the solutions were allowed to cool, quantitatively transferred into 25 mL volumetric flasks, and diluted to volume with ultrapure water (18.2 MΩ·cm).

Elemental analysis was performed using an iCAP 7000 Series ICP–OES spectrometer (Thermo Fisher Scientific, Milan, Italy), equipped with a charge injection device (CID) detector operating in axial plasma configuration. Instrument calibration was carried out using ISO-certified multi-element standard solutions at concentrations ranging from 0.01 to 5 mg/L.

The instrumental parameters were adjusted to ensure optimal analytical performance, including: RF power of 1150 W, plasma gas flow rate of 12 L/min, auxiliary gas flow of 0.5 L/min, nebulizer gas flow of 0.7 L/min, and sample uptake rate of 1.0 mL/min. Emission wavelengths were selected to minimize spectral interferences and were set at 238.204 nm (Fe), 213.856 nm (Zn), 324.754 nm (Cu), 257.610 nm (Mn), 221.647 nm (As), 228.802 nm (Cd), 220.353 nm (Pb), 221.164 nm (Ni), and 267.716 nm (Cr).

All determinations were performed on three independently prepared film samples. Limits of detection (LOD) and limits of quantification (LOQ) were estimated based on three and ten times (3σ and 10σ) the standard deviation of the blank signal, respectively. Although certified reference materials (CRMs) were not available for this specific film matrix, analytical reliability was ensured through recovery studies, reagent blank analyses, repeated measurements, and calibration verification according to the validated analytical protocol. Quality assurance and quality control procedures included reagent blank analyses, calibration verification, recovery assessment, and analysis of independently prepared replicate samples. Reagent blanks were processed with each digestion batch to monitor potential contamination. Method accuracy was evaluated through recovery studies, whereas precision was assessed from repeated measurements and expressed as relative standard deviation (RSD). The analytical performance parameters are summarized in Table 3.

### 3.5. Sample Preparation and Purification for HPLC–MS/MS Analysis

Six subsamples (TQ1–TQ6) were collected from spatially distinct regions of the cast film surface, covering both central and peripheral areas along the casting direction, to account for potential spatial variability and to obtain a representative assessment of compositional homogeneity. The number of subsamples was selected to provide adequate spatial coverage of the film while keeping the analytical workload compatible with triplicate determination of each subsample (*n* = 3), thus yielding eighteen independent determinations per compound. Film homogeneity was subsequently evaluated from the dispersion of the quantified concentrations across the six subsamples (Section 2.3.1).

An aliquot (10 mg) of each subsample was transferred into a microcentrifuge tube and suspended in 1 mL of distilled water. The suspension was homogenized by vortex mixing for 1 min, followed by ultrasonic-assisted extraction for 15 min. After extraction, samples were centrifuged at 6000 rpm for 10 min; the supernatant was collected, diluted tenfold, and subsequently purified using a mixture of phosphate buffer solution (50 mM, pH 3) and methanol (90:10, *v*/*v*).

Sample clean-up was performed by solid-phase extraction (SPE) using Strata XL cartridges (330 mg, 1 mL; Phenomenex, Torrance, CA, USA). Before sample loading, cartridges were activated using methanol and then conditioned with a mixture of phosphate buffer solution (50 mM, pH 3) and methanol (90:10, *v*/*v*). Diluted extracts were loaded onto the cartridges, which were subsequently washed with acidified water (pH 3) to remove polar interfering compounds. Analytes were finally eluted with methanol. The collected eluates were transferred to autosampler vials for subsequent HPLC–MS/MS analysis.

### 3.6. HPLC–MS/MS Targeted Analysis of Polyphenols

Targeted determination of polyphenols incorporated into the chitosan–pomegranate peel films was performed using a previously validated LC–MS/MS method with minor modifications [57]. Briefly, an Acquity H-Class chromatographic system (Waters, Milford, MA, USA) coupled with a QTrap 4500 mass spectrometer (Sciex, Toronto, ON, Canada) was employed. Each sample extract was diluted with 1 mL of phosphate buffer (50 mM)/MeOH (9:1 *v*/*v*), and the resulting solution was subjected to Solid-Phase Extraction (SPE) purification. A Strata XL cartridge (330 mg, 1 mL) from Phenomenex (Torrance, CA, USA) was used for the clean-up step, and the resulting extract was analysed by HPLC-ESI-MS/MS operating in negative electrospray ionization mode. For chromatographic separation, an ACE Excel 2 C18-PFP 2.0 µm (100 × 2.1 mm) column was employed; H_2_O 0.5% acetic acid (phase A) and ACN (phase B) were used as mobile phases. The gradient was programmed as follows: 5% B for 0.3 min; linear increase to 30% B in 1.80 min; further linear increase to 60% B in 3.70 min; ramp to 100% B in 4.45 min with linear increase; 0.25 min isocratic at 99% B; return to initial conditions (5% B) in 2 min. The column operated at a flow rate of 0.6 mL/min, the injection volume was set at 5 µL, and the column oven was set at 40 °C. Multiple Reaction Monitoring (MRM) acquisition mode was used for detection and quantification of 36 analytes; for each phenolic compound, at least two MRM transitions were monitored, each being carefully optimized by injection of the corresponding analytical standard. Data collection and processing were performed with Analyst 1.7.3 software and quantification with Multiquant 3.0.3 software, both from Sciex. The selected ions and the corresponding HPLC–MS/MS parameters are reported in Table 9.

For all analytes, calibration curves were constructed using seven concentration levels (0.025–50 ng mL^−1^) analyzed in triplicate, providing determination coefficients (R^2^) ≥ 0.995, with most compounds ≥ 0.999. Recoveries ranged from 45% to 98%, and the matrix effect (ion suppression), assessed by post-column infusion, remained below 16% for all analytes. Intraday repeatability and interday intermediate precision, expressed as relative standard deviation (RSD), were ≤10%, and validation was performed in accordance with FDA guidelines, with limits of quantification (LOQ) between 0.0004 and 0.06 ng mg^−1^. Individual limits of detection (LOD) and quantification (LOQ) were determined at signal-to-noise ratios of 3:1 and 10:1, respectively. The individual linearity range, R^2^, LOD, LOQ, recovery, and matrix-effect values for the twelve compounds quantified in the present study are summarized in Table 10 (LOD and LOQ are expressed in ng mg^−1^, numerically equivalent to µg g^−1^).

Ellagic acid is included in the group’s validated targeted panel [58], whereas delphinidin-3-glucoside was added for the present application. For both analytes, the MRM transitions were optimized, and the corresponding validation parameters were determined in-house following the same protocol.

### 3.7. Untargeted HPLC–HRMS Analysis

Untargeted high-resolution mass spectrometric analysis was performed using a Vanquish UHPLC system (Thermo Fisher Scientific, Bremen, Germany) coupled to an Orbitrap IQ-X hybrid mass spectrometer equipped with a heated electrospray ionization (H-ESI) source. The analytical workflow was adapted from previously reported approaches [57] and optimized to account for the specific sample matrix and the presence of phenolic compounds in the biofilms.

For chromatographic separation, the same experimental conditions were used as for target analysis: column, mobile phases, and gradient were the same in both methods.

Accurate mass spectra were acquired in both positive and negative ionization modes over an *m*/*z* range of 100–1000 at a resolving power of 60,000 FWHM. HESI source parameters were set according to the manufacturer’s recommendations, including an ion transfer tube temperature of 325 °C, a source heater temperature of 300 °C, and a spray voltage of 2.5 kV. Nitrogen was used as sheath and auxiliary gas. External mass calibration was performed prior to each analytical sequence using Pierce™ FlexMix Calibration Solution (Thermo Fisher Scientific, Bremen, Germany), while fluoranthene was employed as an internal EASY-IC calibrator to ensure mass accuracy within 5 ppm throughout data acquisition.

Raw data were processed using Freestyle 1.8 software, and feature extraction and annotation were performed with Compound Discoverer™ 3.3 (Thermo Fisher Scientific). Tentative identification of detected features was achieved by comparing experimental exact masses and isotopic distribution patterns with theoretical values and reference databases.

Putative identification of the detected features was performed within Compound Discoverer 3.3 following a stepwise annotation workflow. For each feature, the molecular formula was assigned from the accurate mass of the deprotonated/protonated molecule and its isotopic pattern, applying a mass-error threshold of ±5 ppm and an isotopic-pattern (spectral) fit ≥ 85%. Candidate structures matching each molecular formula were retrieved by database searching against ChemSpider, and the resulting annotations were cross-checked against literature data on pomegranate-peel phenolics and ellagitannin chemistry, retaining only candidates consistent with the known phytochemical profile of the matrix and with the observed chromatographic behavior. Following the communicating-confidence framework of Schymanski et al. [59], the detected features are reported as putative identifications at confidence Level 2–3 (probable structure/tentative candidate). Isomeric features that could not be discriminated in the absence of authentic standards are reported at Level 3, whereas annotations supported by diagnostic literature evidence are reported at Level 2. No reference standards were available for these compounds, and no quantification was provided.

### 3.8. Microbiological Analysis

Microbiological analyses were carried out on the edible films in their original form and on *Pecorino Romano PDO* samples with and without film coating. Total viable count (TVC) was determined in accordance with the official UNI EN ISO 4833-1 [60] method for dairy matrices, whereas yeasts and molds were enumerated following UNI EN ISO 21527-2:2008 [61]. Enterobacteriaceae were determined using Violet Red Bile Glucose Agar (VRBG) according to standard microbiological procedures.

A commercial *Pecorino Romano PDO* cheese (net weight 500 g) was purchased from a local grocery store and aseptically divided into subsamples under a Class II biological safety cabinet. Three experimental groups were prepared, each consisting of three independent biological replicates: (i) edible film alone, (ii) uncoated *Pecorino Romano PDO* cheese (control), and (iii) *Pecorino Romano PDO* cheese coated with the edible film. Cheese samples were aseptically wrapped with the edible film using sterile forceps inside a Class II laminar-flow biological safety cabinet to ensure complete surface coverage and minimize the risk of external contamination.

Each cheese sample weighed approximately 10 g and was individually placed in sterile 90 mm Petri dishes under aerobic conditions, without vacuum or modified packaging atmosphere. The edible films alone were stored under identical conditions. All samples were maintained in a laboratory refrigerator at 4.5 °C with a relative humidity of 45 ± 7% throughout the 45-day storage period. Microbiological analyses were performed at four sampling times (T0–T3).

At each sampling time, 300 mg of sample were aseptically transferred into sterile tubes containing 2700 μL of buffered peptone water (BPW) and homogenized for 1 min using a high-speed vortex mixer. Tenfold serial dilutions were prepared and plated on selective media as follows: total viable cell count on Plate Count Agar (PCA), incubated at 30 °C for 72 h; yeasts and moulds on Sabouraud Dextrose Agar supplemented with chloramphenicol, incubated at 25 °C for 5 days; and Enterobacteriaceae on Violet Red Bile Glucose Agar (VRBG), incubated at 37 °C for 24 h.

After incubation, colonies were counted, and microbiological data were expressed as log CFU/g. All determinations were performed in triplicate, and results are expressed as mean ± standard deviation (SD) of three independent biological replicates.

### 3.9. Statistical Analysis

Data are presented as mean ± standard deviation (SD) of measurements performed in triplicate. For microbiological analyses, statistical comparisons between coated and uncoated cheese at each refrigerated storage time were performed using Welch’s *t*-test, which was selected because it is appropriate for comparison between two independent groups, assuming homogeneity of variance. Differences were considered statistically significant at *p* < 0.05. Statistical analyses were carried out using GraphPad Prism 10 (GraphPad Software, San Diego, CA, USA).

## 4. Conclusions

The present study demonstrated that the patented chitosan-based edible biofilm, enriched with pomegranate peel extract, essential oils, and propolis, represents a promising multifunctional coating for refrigerated dairy products. The integrated analytical approach, combining ICP–OES, targeted HPLC–MS/MS, untargeted HPLC–HRMS, mechanical characterization, SEM observations, and microbiological evaluation, confirmed the chemical safety, structural integrity, and antimicrobial performance of the developed material. Total quantified polyphenols ranged from 88.20 to 137.38 μg/g dry weight, with ellagic acid identified as the predominant phenolic compound (90.78 μg/g dry weight). Heavy metals were below the analytical quantification limits, supporting compliance with food-contact safety requirements. During refrigerated storage, coated Pecorino Romano PDO cheese consistently exhibited lower yeast and mold counts than the untreated control, with reductions approaching 0.9 log CFU/g at intermediate storage times, while Enterobacteriaceae remained below the detection limit throughout the storage period.

These findings demonstrate that the incorporation of pomegranate peel extract into a chitosan-based matrix provides an effective strategy for developing sustainable active packaging materials while simultaneously promoting the valorization of agro-industrial by-products within a circular economy framework. The combination of targeted and untargeted chemical characterization, elemental safety assessment, mechanical evaluation, and longitudinal microbiological monitoring provides a comprehensive evaluation of the patented biofilm under realistic refrigerated storage conditions.

Although the developed edible coating exhibited promising physicochemical, mechanical, and antimicrobial performance, additional studies are warranted to further characterize its technological properties. Future investigations should include the evaluation of antioxidant activity, barrier properties, migration behavior of bioactive compounds, and the possible effects of the coating on the visual appearance of cheese by means of instrumental color measurements (CIELab) and sensory evaluation. These complementary analyses will provide further information on consumer acceptance and support the technological validation of the developed edible biofilm for industrial food-packaging applications.

## 5. Patents

The authors declare that Andrea Salvo and Valentina Mangano have #Patent N° 102021000013913 licensed to the Ministry of Business and Made in Italy. The other authors declare that they have no financial interests or personal relationships that could have influenced the work reported in this article.

## Figures and Tables

**Table 1:** Representative recent studies on chitosan-based edible films and coatings incorporating bioactive compounds for cheese preservation.

Year	Chitosan-Based System	Bioactive Agent/ Functional Component	Cheese Application	Main Findings	Research Gaps/ Limitations	References
2016	Chtosan/CMC/ZnO film	Zinc oxide nanoparticles	Egyptian soft white cheese	Extended shelf life and improved microbiological stability of soft cheese.	Use of inorganic nanomaterials raises potential regulatory and migration concerns.	Youssef et al., 2016 [15]
2018	Chitosan coating	Lysozyme or natamycin	Halloumi cheese	Improved microbiological and sensory quality, extending shelf life even under reduced-salt brining conditions.	Bioactive agents mainly provided antimicrobial activity, while antioxidant properties were not investigated.	Mehyar et al., 2018 [16]
2018	Nanostructured chitosan film	Monolaurin	Ultrafiltered white cheese	Antimicrobial activity against *Listeria monocytogenes*, improving microbiological safety.	Study focused on a single pathogen, with limited evaluation of overall shelf-life.	Lotfi et al., 2018 [17]
2020	Edible chitosan coating	Natamycin	Iranian ultrafiltered cheese	Improved microbiological, physicochemical, and sensory quality while effectively controlling fungal contamination.	Mainly focused on antifungal activity, with limited comparison to plant-derived bioactive extracts.	Nottagh et al., 2020 [18]
2021	Chitosan/whey protein coating	TiO_2_ nanoparticles and *Zataria multiflora* essential oil	UF-Feta-type cheese	Reduced total microbial load, coliforms, yeasts, molds, and *Listeria monocytogenes*, thereby extending shelf life.	Complex formulation containing nanoparticles and essential oil, with possible sensory and regulatory concerns.	Gohargani et al., 2021 [19]
2023	Chitosan–gelatin film/coating	Eugenol and oregano essential oil	Fresh cheese	Enhanced antimicrobial and antioxidant properties, improving fresh cheese preservation.	Multicomponent system; essential oils may influence sensory characteristics.	Reyes-Méndez et al., 2023 [20]
2024	Chitosan film	Rosemary extract	Cheese	Extended shelf life and improved microbiological quality through incorporation of a plant-derived antioxidant and antimicrobial extract.	Evaluation is limited to a single plant extract; further validation on different dairy matrices is required.	Ghasemian et al., 2024 [21]
2024	Chitosan–furcellaran film	LL-37 and RW4 antimicrobial peptides	Provolone cheese	Improved microbiological safety and cheese quality through antimicrobial biopolymeric films.	High cost of antimicrobial peptides and the need for industrial-scale validation.	Pluta-Kubica et al., 2024 [22]

**Table 2:** Health-based guidance values for arsenic, cadmium, mercury, and lead expressed as tolerable weekly intake (TWI) per kilogram of body weight, according to JECFA and FAO/WHO recommendations. These values are reported for toxicological reference purposes only and are not directly applicable to the elemental content of food-contact materials.

Element	Dose (mg/Week)	References
As	0.015	JECFA 1988 [38]
Cd	0.007	JECFA 1993 [36]
Hg	0.005	FAO/WHO (JECFA 1993) [37]
Pb	0.025	JECFA 1993 [36]

**Table 3:** Analytical performance of the ICP-OES method.

Element	LOD (ng/g)	LOQ (ng/g)	R^2^	Accuracy (%)	Precision (RDS) %, *n* = 10
As	0.011	0.045	0.99992	102.61	3.3
Cd	0.023	0.081	0.99989	110.99	3.8
Hg	0.016	0.040	0.99991	90.78	2.8
Pb	0.030	0.097	0.99987	96.50	3.5

**Table 4:** Elemental concentrations determined in the chitosan–pomegranate peel biofilm by ICP-OES.

Element	Result (ng/g)	LOD (ng/g)
As	<LOD	0.011
Cd	<LOD	0.023
Hg	<LOD	0.016
Pb	<LOD	0.030

**Table 5:** Targeted HPLC–MS/MS polyphenols identified and quantified in biofilm samples. Results are expressed as μg/g dry weight (dw) and reported as mean ± SD (*n* = 3). Total quantified polyphenols represent the sum of the twelve detected compounds.

Compound	TQ1	TQ 2	TQ 3	TQ 4	TQ 5	TQ 6
Gallic acid	20.420 ± 1.471	20.579 ± 1.132	20.420 ± 1.756	20.300 ± 1.238	19.768 ± 0.949	28.800 ± 2.621
Syringic acid	0.028 ± 0.002	0.021 ± 0.002	0.028 ± 0.001	0.021 ± 0.001	0.017 ± 0.001	0.036 ± 0.003
Apigenin	0.056 ± 0.003	0.056 ± 0.004	0.056 ± 0.003	0.018 ± 0.002	0.037 ± 0.002	0.049 ± 0.005
Epicatechin	0.328 ± 0.017	0.370 ± 0.025	0.328 ± 0.015	0.365 ± 0.028	0.285 ± 0.023	0.422 ± 0.025
Myricetin	0.032 ± 0.002	0.031 ± 0.002	0.032 ± 0.003	0.030 ± 0.001	0.021 ± 0.002	0.030 ± 0.002
Chlorogenic acid	0.044 ± 0.002	0.047 ± 0.003	0.044 ± 0.003	0.047 ± 0.002	0.044 ± 0.003	0.047 ± 0.004
Quercetin hexoside	11.150 ± 0.702	13.341 ± 0.654	11.150 ± 0.903	13.160 ± 0.737	10.689 ± 0.791	15.398 ± 1.047
Rutin	0.240 ± 0.016	0.352 ± 0.018	0.240 ± 0.020	0.348 ± 0.016	0.221 ± 0.018	0.299 ± 0.018
Protocatechuic acid	0.354 ± 0.019	0.403 ± 0.028	0.354 ± 0.027	0.397 ± 0.019	0.326 ± 0.028	0.478 ± 0.030
Naringenin	0.098 ± 0.005	0.086 ± 0.006	0.098 ± 0.008	0.084 ± 0.004	0.082 ± 0.008	0.094 ± 0.006
Ellagic acid	66.500 ± 3.190	65.365 ± 4.050	66.500 ± 4.990	64.480 ± 3.480	55.994 ± 4.540	90.780 ± 3.900
Delphinidin-glucoside	0.751 ± 0.038	0.864 ± 0.055	0.751 ± 0.062	0.852 ± 0.041	0.718 ± 0.054	0.948 ± 0.056
Total quantified polyphenols	100.001	101.515	100.001	100.102	88.202	137.381

**Table 6:** Untargeted HPLC–HRMS profiling of the phenolic compounds detected in the chitosan–pomegranate peel biofilm. Annotations are putative (confidence Level 2–3) and were obtained from accurate mass, isotopic pattern, ChemSpider matching, and literature comparison; no quantification was performed. Theoretical *m*/*z* values refer to the indicated ion; mass errors are reported in ppm. The corresponding extracted ion chromatograms are shown in Figure S1.

Tentative Compound	Molecular Formula	Ion	Theoretical *m*/*z*	Experimental *m*/*z*	Δ (ppm)	RT (min)	Max. Peak Area	ChemSpider Hits (n)
Flavogallol (isomer I) *	C_21_H_8_O_12_	[M+H]^+^	453.0089	453.0098	2.1	3.64	11.3 × 10^6^	27
Ellagic acid glucoside (isomer I) *	C_20_H_16_O_13_	[M−H]^−^	463.0518	463.0523	1.1	4.05	10.7 × 10^6^	63
Methylpyrogallol sulfate	C_7_H_8_O_6_S	[M+H]^+^	221.0114	221.0108	−3.0	0.99	8.8 × 10^6^	6
8-Hydroxyluteolin 8-glucoside-3′-sulfate	C_21_H_20_O_15_S	[M−H]^−^	543.0450	543.0424	−4.9	3.28	7.4 × 10^6^	22
2-O-*p*-Coumaroylhydroxycitric acid	C_15_H_14_O_10_	[M−H]^−^	353.0514	353.0519	1.4	2.54	6.2 × 10^6^	4
Sanguisorbic acid dilactone	C_21_H_10_O_13_	[M+H]^+^	471.0194	471.0205	2.2	3.77	5.9 × 10^6^	25
Castanin	C_34_H_24_O_22_	[M−H]^−^	783.0687	783.0690	0.4	3.63	4.7 × 10^6^	11
Flavogallol (isomer II) *	C_21_H_8_O_12_	[M +H]^+^	453.0089	453.0092	0.8	3.28	3.4 × 10^6^	26
Ellagic acid 2-rhamnoside	C_20_H_16_O_12_	[M−H]^−^	447.0569	447.0574	1.2	4.51	3.2 × 10^6^	71
Catechin 7-sulfate	C_15_H_14_O_9_S	[M−H]^−^	369.0286	369.0274	−3.1	2.01	3.0 × 10^6^	31
Ellagic acid glucoside (isomer II) *	C_20_H_16_O_13_	[M−H]^−^	463.0518	463.0524	1.2	4.32	2.8 × 10^6^	62
Flavogallol (isomer III) *	C_21_H_8_O_12_	[M+H]^+^	453.0089	453.0097	1.8	4.12	2.6 × 10^6^	26
1,6-bis-O-galloyl-*β*-*D*-glucose	C_20_H_20_O_14_	[M−H]^−^	483.0780	483.0784	0.8	2.82	1.8 × 10^6^	91
Diellagilactone	C_28_H_10_O_16_	[M−H]^−^	600.9896	600.9905	1.5	4.44	1.7 × 10^6^	2
6-Methoxyluteolin 7-glucuronide	C_22_H_20_O_13_	[M−H]^−^	491.0831	491.0840	1.9	4.78	1.6 × 10^6^	100
Cymorcin diglucoside	C_22_H_34_O_12_	[M−H]^−^	489.1978	489.1984	1.4	4.29	1.0 × 10^6^	124

* Compounds sharing the same molecular formula and accurate mass, proposed as isomeric forms (resolved chromatographically; see Figure S1). Mass error (ppm) = (experimental − theoretical *m*/*z*)/theoretical *m*/*z* × 10^6^. All annotations fall within ±5 ppm. ChemSpider hits (n) = number of candidate structures returned by ChemSpider for the assigned molecular formula.

**Table 7:** Microbial counts in edible biofilm (in its original form) during 45 days of storage (T0–T3). Values are expressed as mean ± standard deviation (*n* = 3).

Storage Time (h)	Sampling Time	TVC (30 °C) (log_10_ CFU/g)	Yeasts and Molds (log_10_ CFU/g)
0	T0	0.00 ± 0.00	0.00 ± 0.00
360	T1	2.17 ± 0.12	0.95 ± 0.00
720	T2	2.07 ± 0.16	0.72 ± 0.01
1080	T3	1.54 ± 0.16	0.00 ± 0.00

**Table 8:** Microbial counts of uncoated (control) and coated Pecorino Romano PDO cheese during 45 days of refrigerated storage (T0–T3). Values are expressed as mean ± standard deviation (*n* = 3). Statistical comparison between coated and control samples at each storage time was performed using Welch’s *t*-test.

Storage Time (h)	Sampling Time	TVC (30 °C) (log_10_ CFU/g) Control	TVC (30 °C) (log_10_ CFU/g) Coated	Log Reductio TVC	*p*-Value TVC	Yeasts & Molds (log_10_ CFU/g) Control	Yeasts & Molds (log_10_ CFU/g) Coated	Log Reductio (Fungi)	*p*-Value Yeast and Molds
0	T0	3.48 ± 0.00	3.51 ± 0.43	0.00	0.912	3.52 ± 0.07	3.39 ± 0.16	0.13	0.281
360	T1	6.45 ± 0.02	6.23 ± 0.04	0.22	0.0037 **	2.43 ± 0.13	1.56 ± 0.42	0.87	0.060
720	T2	6.04 ± 0.06	5.46 ± 0.65	0.58	0.257	3.01 ± 0.14	2.26 ± 0.25	0.75	0.0197 *
1080	T3	6.80 ± 0.08	6.13 ± 0.07	0.67	0.0006 ***	3.09 ± 0.13	2.89 ± 0.06	0.20	0.099

* *p* < 0.05; ** *p* < 0.01; *** *p* < 0.001.

**Table 9:** Retention times (R_T_), optimized multiple reaction monitoring (MRM) transitions, and mass spectrometric parameters of polyphenolic compounds in chitosan-pomegranate peel edible film analyzed by HPLC–MS/MS. Precursor ion *m*/*z* values (Q1) are reported as [M−H]^−^, and product ion *m*/*z* values (Q3) are also reported. They agree with data reported by Oliva et al., 2021 [57].

N°	Compound	R_T_ (min)	Q_1_ (amu)	DP (V)	EP (V)	Q_3_ (amu)	CE (V)	CXP (V)
1	Delfinidin-3-glucoside	1.55	464.9	−90.00	−8.00	302.9	−40.00	−10.00
						391.2	−45.00	−12.00
2	Gallic acid	2.05	168.9	−70.00	−10.50	124.9	−21.00	−9.00
						96.9	−26.00	−6.00
3	Ellagic acid	2.51	301.0	−80.00	−7.00	145.0	−40.00	−7.00
						283.9	−45.00	−9.00
4	Hydroxytyrosol	3.22	152.9	−75.00	−7.00	122.9	−22.00	−8.00
						104.6	−30.00	−7.00
5	Protocatechuic acid	3.95	152.8	−64.00	−12.00	108.9	−23.00	−6.00
						53	−34.00	−4.00
6	Epigallocatechin	4.53	305.0	−98.00	−6.50	136.9	−36.00	−10.00
						166.9	−28.50	−6.50
7	3,4-Hydroxybenzoic acid	4.60	136.9	−12.00	−9.00	89.0	−15.50	−6.00
						66.0	−30.00	−10.00
8	Tyrosol	4.61	137.0	−65.00	−9.00	119.0	−21.00	−8.50
						106.9	−23.00	−5.50
9	Chlorogenic acid	4.64	353.1	−60.00	−7.00	190.9	−25.50	−6.50
						160.9	−35.00	−6.00
10	Epicatechin	4.73	288.9	−85.00	−8.00	245.0	−24.00	−9.00
						108.9	−38.50	−8.00
11	Caffeic acid	5.05	179.0	−60.00	−10.00	135.0	−22.00	−8.00
						107.0	−32.00	−10.00
12	Vanillic acid	5.06	167.1	−49.00	−11.00	107.9	−28.50	−5.00
						151.9	−19.50	−6.50
13	Catechin	5.10	289.0	−95.00	−5.00	245.0	−25.00	−8.00
						108.9	−32.50	−9.00
14	Epigallocatechin gallate	5.19	457.2	−10.00	−7.00	168.8	−20.00	−17.00
						124.9	−40.00	−17.00
15	Syringic acid	5.21	196.9	−57.00	−11.00	181.9	−19.00	−6.00
						120.9	−22.00	−8.00
16	Orientin	5.35	447.1	−105.00	−11.00	356.9	−29.00	−11.00
						297.0	−43.00	−11.00
17	Rutin	5.52	609.2	−100.00	−6.00	300.9	−45.00	−11.00
						254.7	−70.00	−9.00
18	*p*-Coumaric acid	5.67	162.8	−60.00	−5.00	119.0	−20.00	−9.00
						116.7	−42.00	−9.00
19	Hyperoside	5.68	462.9	−110.00	−5.00	300.9	−48.00	−10.00
						299.9	−40.00	−11.00
20	Isoquercitin	5.69	463.0	−100.00	−9.00	300.9	−33.00	−11.00
						270.9	−58.00	−10.00
21	Ferulic acid	5.96	192.8	−63.00	−11.00	134.0	−21.00	−8.00
						177.8	−18.00	−6.00
22	Hesperidin	6.01	609.1	−130.00	−4.50	300.9	−38.00	−11.00
						286.2	−60.00	−9.00
23	Rosmarinic acid	6.20	359.0	−78.00	−4.50	160.8	−24.00	−7.00
						197.0	−24.50	−6.50
24	Oleuropein	6.20	539.2	−55.00	−10.00	275.0	−32.00	−9.00
						377.1	−24.00	−9.00
25	*o*-Coumaric acid	6.31	163.0	−50.00	−5.00	119.0	−19.00	−8.00
						117.0	−33.00	−7.00
26	Synapic acid	6.35	222.9	−75.00	−11.00	164.0	−23.00	−6.00
						208.0	−22.00	−7.00
27	Myricetin	6.42	316.9	−115.00	−5.00	150.9	−34.00	−6.00
						136.9	−36.00	−9.50
28	Luteolin	6.79	284.9	−100.00	−8.00	150.9	−35.00	−11.00
						199.0	−35.00	−6.00
29	Quercetin	6.84	300.9	−94.00	−10.00	151.0	−30.00	−6.00
						178.8	−26.00	−6.00
30	*trans*-Cinnamic acid	6.92	147.2	−50.00	−8.00	102.9	−16.00	−4.00
						77.2	−30.00	−6.00
31	Naringenin	6.95	271.0	−112.00	−5.00	150.9	−25.00	−7.00
						118.8	−37.00	−8.00
32	Isoxanthohumol	7.06	353.1	−114.00	−10.00	233.1	−26.00	−8.00
						189.1	−35.00	−9.00
33	Apigenin	7.19	268.9	−110.00	−4.00	116.9	−50.00	−9.00
						151.0	−34.00	−5.00
34	Diosmetin	7.27	299.1	−90.00	−6.00	255.8	−40.00	−9.00
						150.9	−40.00	−10.00
35	Kaempferol	7.27	285.0	−105.00	−7.00	228.8	−40.00	−9.00
						159.0	−42.00	−6.00
36	Xhanthumol	9.48	353.0	−120.00	−8.00	164.9	−42.00	−9.00
						203.0	−45.00	−8.00

For each analyte, the first transition was used for quantification, and the second transition for confirmation.

**Table 10:** Validation parameters of the targeted HPLC–MS/MS method for the twelve phenolic compounds quantified in the chitosan–pomegranate peel biofilm. Parameters for the ten compounds belonging to the originally validated method are reported from Oliva et al. [57].

Compound	Linearity (ng mL^−1^)	R^2^	LOD (ng mg^−1^)	LOQ (ng mg^−1^)	Recovery (%)	Matrix Effect (%)
Gallic acid	0.025–50	0.996	0.0152	0.0505	54–89	−10 to +13
Protocatechuic acid	0.025–50	0.999	0.0054	0.0181	72–84	−4 to +13
Chlorogenic acid	0.025–50	0.999	0.0007	0.0023	73–88	−14 to +16
Epicatechin	0.025–50	0.998	0.0109	0.0362	65–74	−11 to +13
Syringic acid	0.025–50	0.999	0.0008	0.0028	75–98	−12 to +16
Rutin	0.025–50	0.999	0.0025	0.0084	65–83	−6 to +7
Quercetin hexoside ^a^	0.025–50	0.998	0.0002	0.0008	72–82	−4 to +13
Myricetin	0.025–50	0.995	0.0029	0.0096	45–65	−18 to +3
Naringenin	0.025–50	0.999	0.0017	0.0057	85–96	−6 to +7
Apigenin	0.025–50	0.999	0.0005	0.0015	53–66	−15 to +12
Ellagic acid ^b^	0.025–50	0.999	0.0004	0.0014	58–70	−10 to +8
Delphinidin-3-glucoside ^b^	0.025–50	0.999	0.0008	0.0030	58–70	−12 to +8

^a^ Quantified against quercetin-3-O-hexoside standards (hyperoside/isoquercitrin); the reported values refer to hyperoside. ^b^ Ellagic acid belongs to the group’s validated targeted panel and was quantified in plant matrices in Oliva et al. [58] (global LOQ range 0.0004–0.06 ng mg^−1^); delphinidin-3-glucoside was added for the present application. For both analytes, the individual validation parameters reported here were determined in-house following the same protocol. Calibration range, R^2^, LOD, LOQ, recovery, and matrix-effect values for the ten analytes in rows 1–10 are taken from Oliva et al. [57].

## Data Availability

The original contributions presented in this study are included in the article. Further inquiries can be directed to the corresponding author(s).

## Supplementary Materials

The following supporting information can be downloaded at https://www.mdpi.com/article/10.3390/molecules31152631/s1: Figure S1: Extracted ion chromatograms (XICs) of the phenolic compounds putatively identified in the chitosan–pomegranate peel biofilm by untargeted HPLC–HRMS (Orbitrap IQ-X, full-scan FTMS; sample biofilm). Each trace was extracted at the theoretically accurate mass of the corresponding molecular ion within a ±5 ppm window. For several compounds, multiple chromatographic peaks share the same accurate mass and correspond to positional/structural isomers that could not be unambiguously resolved in the absence of authentic standards. Panels (**a**–**m**) refer to the annotations reported in Table 6.
