# Supplementary material for: Sustainable Active Packaging Based on Chitosan–Pomegranate Peel Biofilm Protects Pecorino Romano PDO Cheese Through Polyphenol-Driven Antioxidant and Antimicrobial Activity
Source: Molecules. 2026 Jul 28;31(15):2631. doi: 10.3390/molecules31152631 (PMC13468628; doi:10.3390/molecules31152631)
Supplement: Supplementary file 1 [file molecules-31-02631-s001.zip › molecules-4389739-supplementary.pdf]

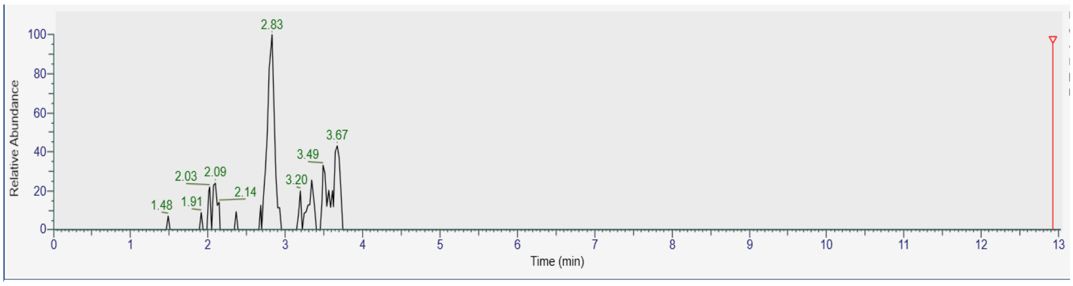

(a) 1,6-bis-O-galloyl-P-D-glucose, $C_{20}H_{20}O_{14}$, $[M{-}H]^-$.

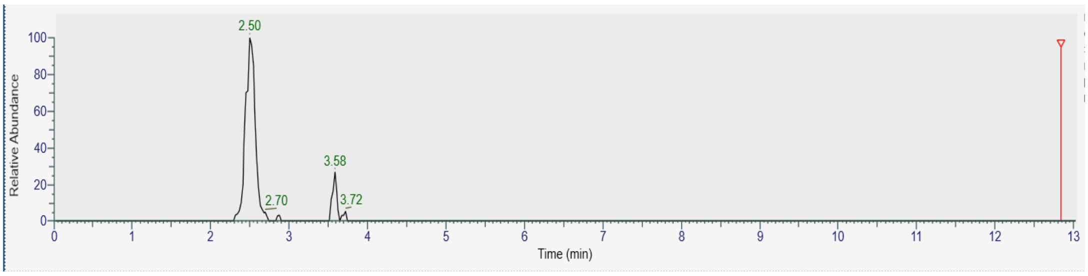

(b) 2-0-*p*-Coumaroylhydroxycitric acid, $C_{15}H_{14}O_{10}$, $[M{-}H]^-$.

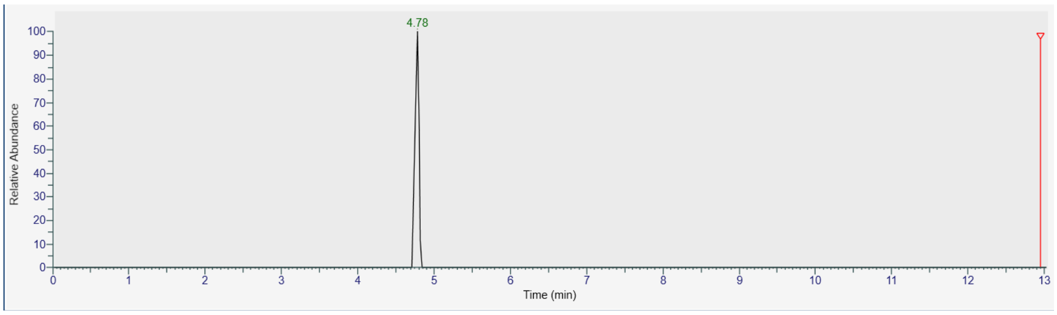

(c) 6-Methoxyluteolin 7-glucuronide, $C_{22}H_{20}O_{13}$, $[M{-}H]^-$.

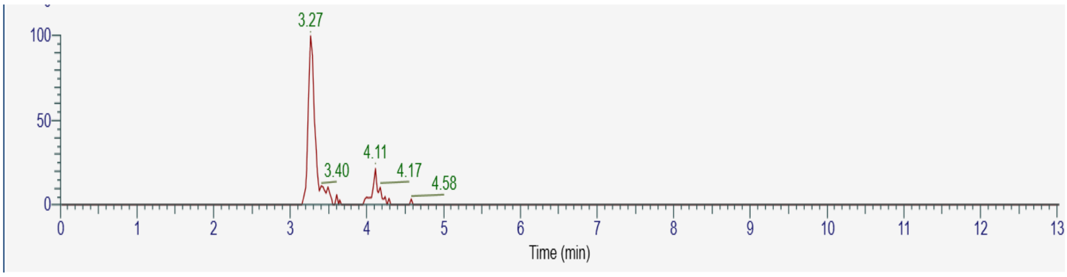

(d) 8-Hydroxyluteolin 8-glucoside-3′-sulfate, $C_{21}H_{20}O_{15}S$, $[M{-}H]^-$.

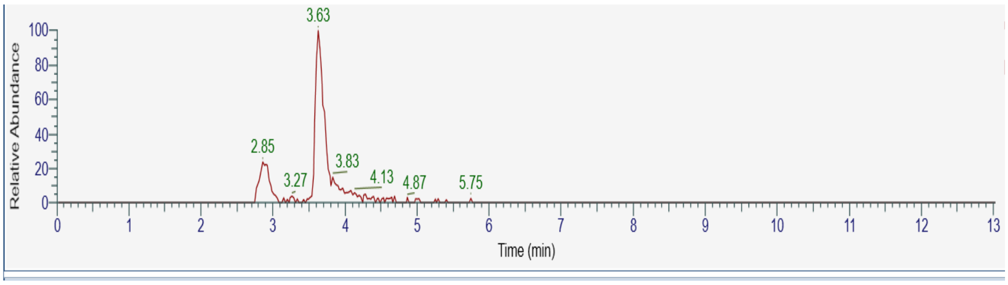

(e) Castanin , $C_{34}H_{24}O_{22}$, $[M{-}H]^-$.

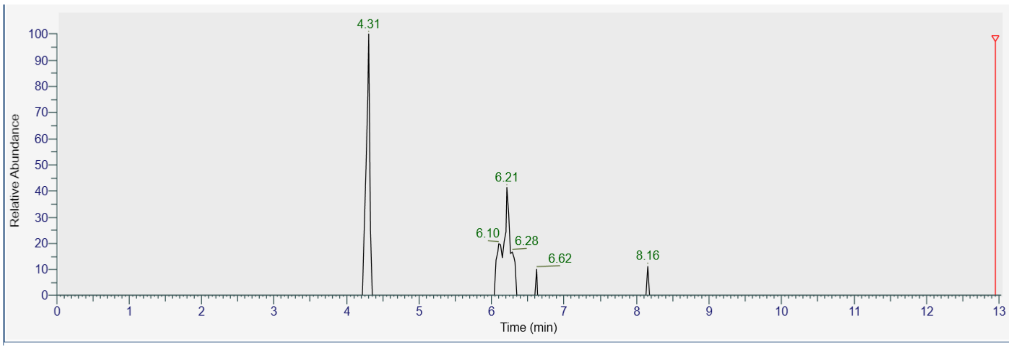

(f) Cymorcin diglucoside, $C_{22}H_{34}O_{12}$, $[M-H]^-$.

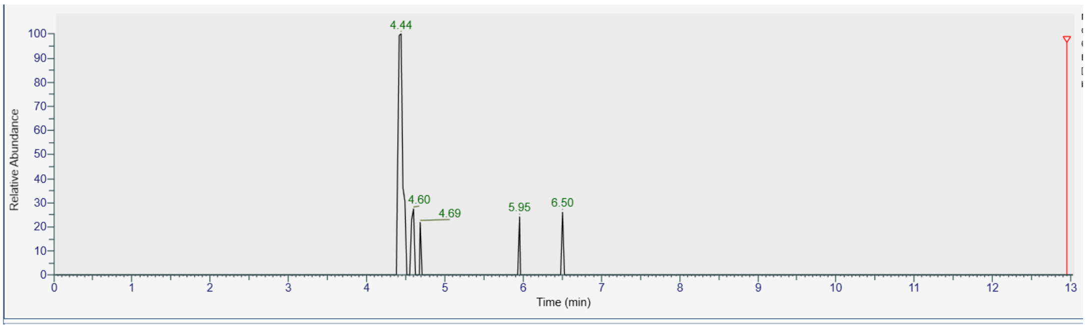

(g) Diellagilactone, $C_{28}H_{10}O_{16}$, $[M-H]^-$.

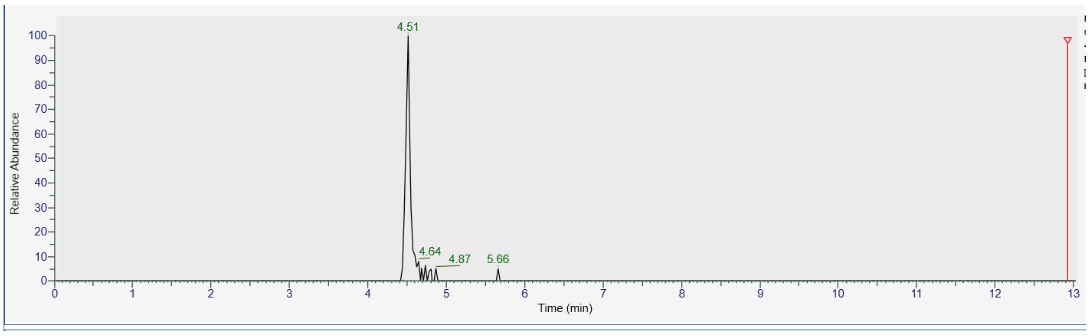

(h) Ellagic acid 2-rhamnoside, $C_{20}H_{16}O_{12}$, $[M-H]^-$.

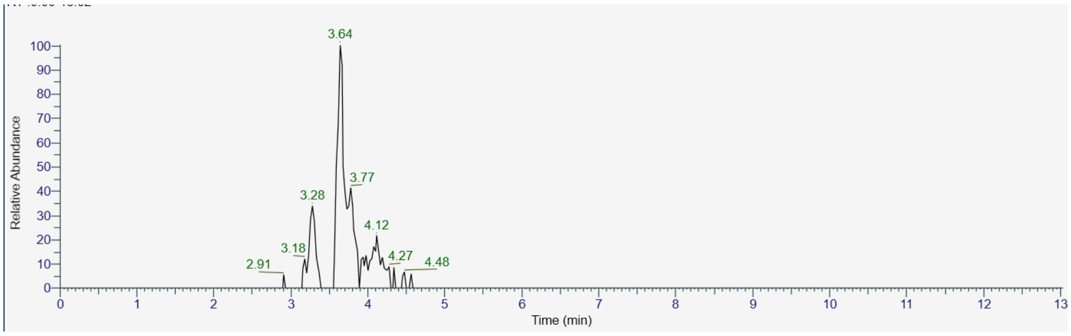

(i) Flavogallol (isomers I–III), $C_{21}H_8O_{12}$, $[M+H]^+$.

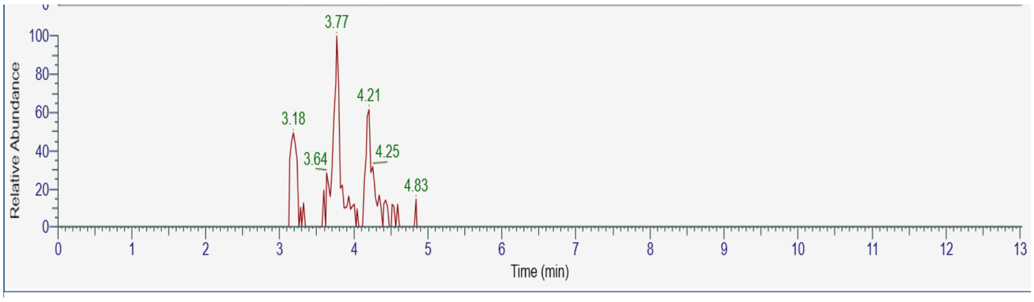

(j) Sanguisorbic acid dilactone, $C_{21}H_{10}O_{13}$, $[M+H]^+$.

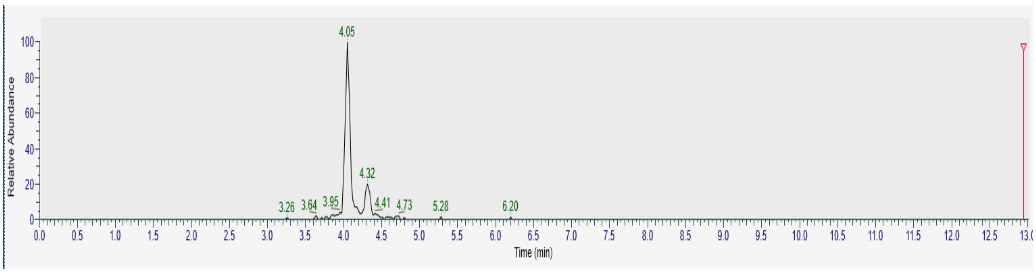

(k) Ellagic acid glucoside (isomers I–II), $C_{20}H_{16}O_{13}$, $[M-H]^-$.

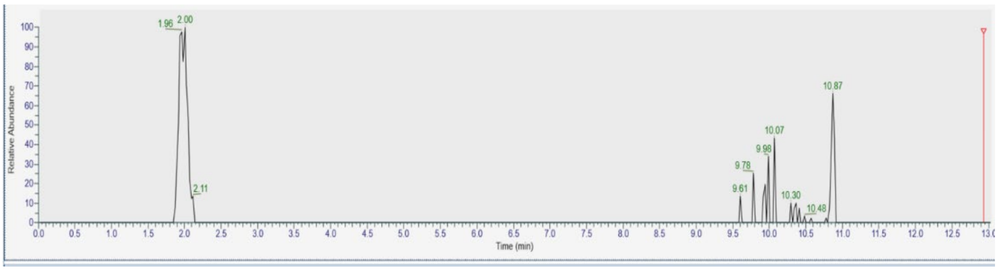

(l) Catechin7-sulfate, C15H14O9S, $[M-H]^-$.

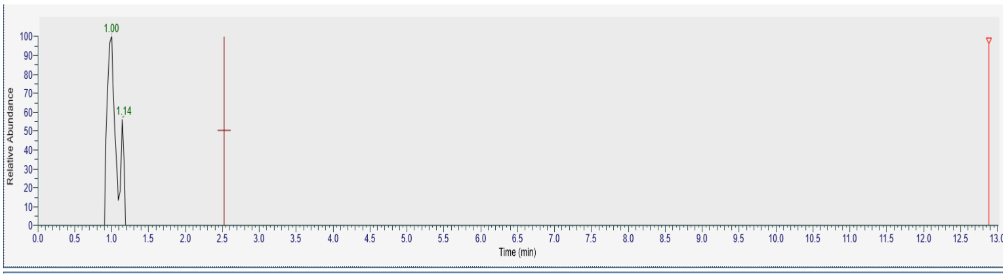

(m) Methylpyrogallol sulfate, C7H8O6 S, [M+H]+.

Figure S1. Extracted ion chromatograms (XICs) of the phenolic compounds putatively identified in the chitosan–pomegranate peel biofilm by untargeted HPLC–HRMS (Orbitrap IQ-X, full-scan FTMS; sample biofilm). Each trace was extracted at the theoretically accurate mass of the corresponding molecular ion within a ±5 ppm window. For several compounds, multiple chromatographic peaks share the same accurate mass and correspond to positional/structural isomers that could not be unambiguously resolved in the absence of authentic standards. Panels **(a–m)** refer to the annotations reported in Table 6.
